# Supplementary material for: Characterization and Dye Decolorization Potential of Two Laccases from the Marine-Derived Fungus Pestalotiopsis sp
Source: Int J Mol Sci. 2019 Apr 15;20(8):1864. doi: 10.3390/ijms20081864 (PMC6515530; doi:10.3390/ijms20081864)
Supplement: Supplementary file 1 [file ijms-20-01864-s001.pdf]

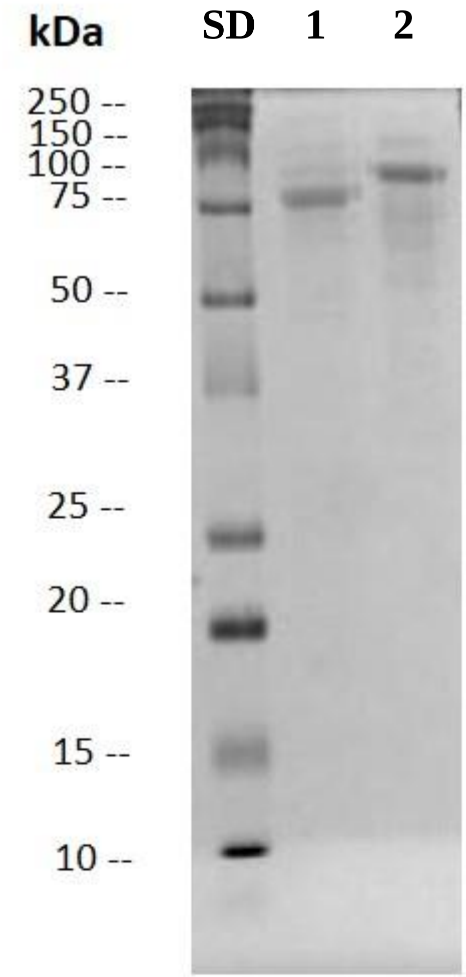

Supplementary Figure 1 : SDS-PAGE analysis of the purified *Ps*Lac1 (lane 1) and *Ps*Lac2 (lane 2). SD are molecular mass standards. Proteins stained with Coomassie blue,

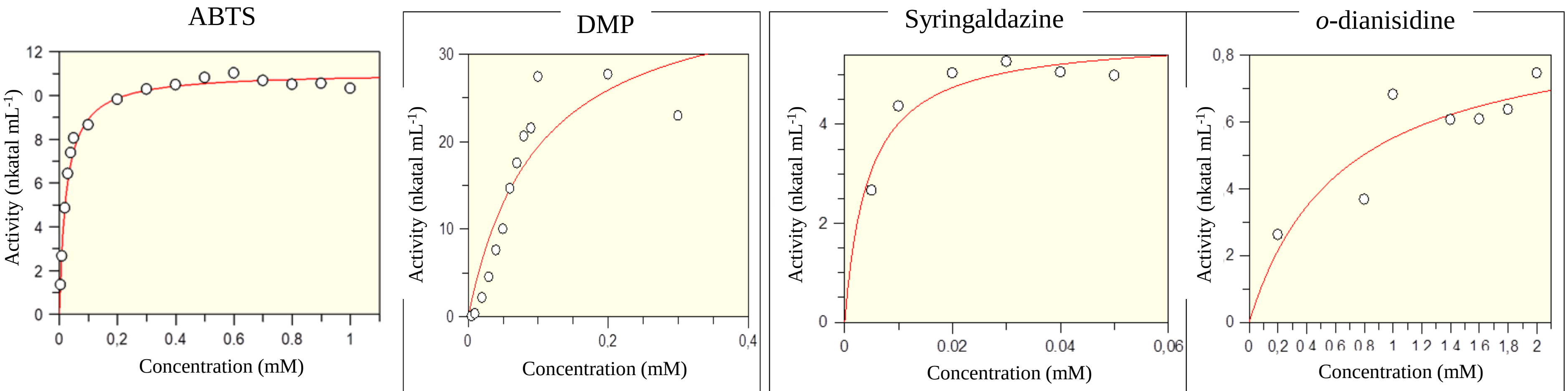

ABTS

Parameter	Value	Std. Error
Vmax	11,0450	0,1471
Km	0,0238	0,0018

DMP

Parameter	Value	Std. Error
Vmax	38,7348	7,7493
Km	0,0995	0,0404

Syringaldazine

Parameter	Value	Std. Error
Vmax	5,7908	0,3514
Km	0,0044	0,0013

o-dianisidine

Parameter	Value	Std. Error
Vmax	0,9098	0,2069
Km	0,6470	0,4441

Supplementary Figure 2 : Kinetic parameters of *Ps*Lac1

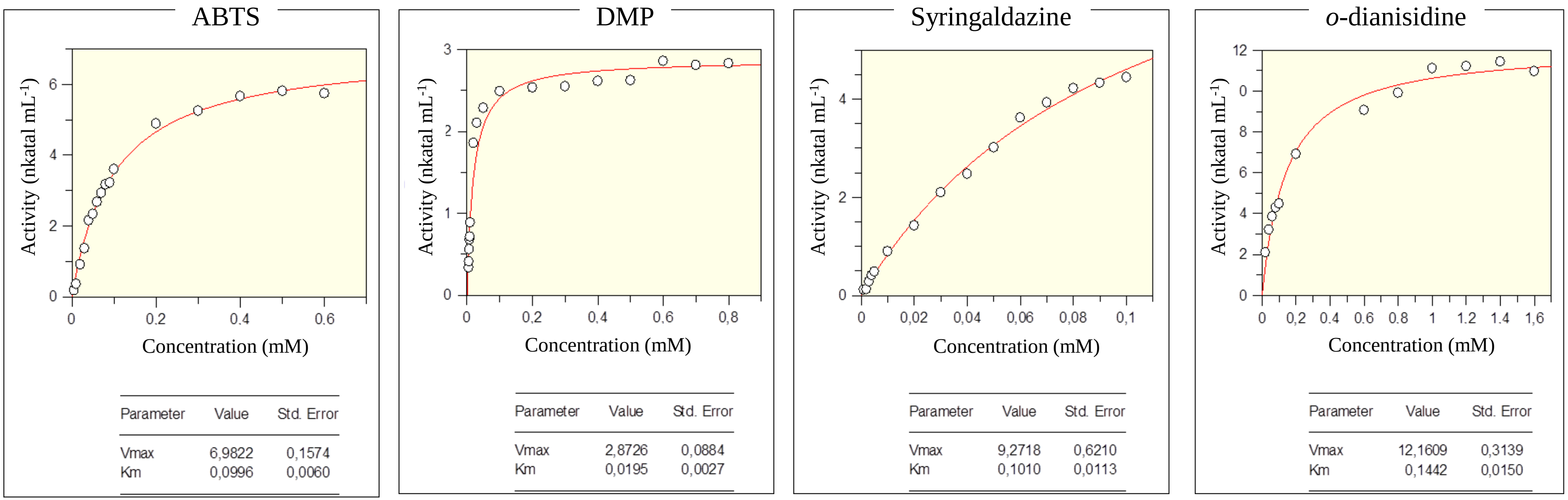

ABTS

Parameter	Value	Std. Error
Vmax	6,9822	0,1574
Km	0,0996	0,0060

DMP

Parameter	Value	Std. Error
Vmax	2,8726	0,0884
Km	0,0195	0,0027

Syringaldazine

Parameter	Value	Std. Error
Vmax	9,2718	0,6210
Km	0,1010	0,0113

o-dianisidine

Parameter	Value	Std. Error
Vmax	12,1609	0,3139
Km	0,1442	0,0150

Supplementary Figure 3 : Kinetic parameters of *Ps*Lac2
